# 2260. Differences in the Prevalence of Inappropriate Antibiotic Prescribing by Payer Type

**DOI:** 10.1093/ofid/ofad500.1882

**Published:** 2023-11-27

**Authors:** Joseph B Ladines-Lim, Kao-Ping Chua, Michael Fischer, Jeffrey A Linder

**Affiliations:** Michigan Medicine, Ann Arbor, Michigan; Michigan Medicine, Ann Arbor, Michigan; Boston University, Boston, Massachusetts; Northwestern University Feinberg School of Medicine, Chicago, IL

## Abstract

**Background:**

Understanding the relationship between inappropriate prescribing and patient insurance type could inform the targeting of antibiotic stewardship initiatives. However, few national data on this topic exist.

**Methods:**

We analyzed the National Ambulatory Medical Care Survey, a nationally representative survey of office-based physician visits. We included all years from 2016 to 2019 except 2017, a year during which the survey was not fielded. We identified visits resulting in an oral antibiotic prescription among children and non-elderly adults who were privately insured, publicly insured, or uninsured. The exposure was safety-net status (publicly insured/uninsured versus privately insured). The outcome was inappropriate antibiotic prescribing, defined as an antibiotic prescription in a visit for which none of the diagnosis codes justified antibiotic use (following the approach of Chua et al, *BMJ* 2019). We assessed the association between safety-net status and inappropriate antibiotic prescribing using linear regression, controlling for patient characteristics, calendar quarter, and specialty. Models accounted for the complex design of the survey and employed designed-based variance estimators.

**Results:**

Of 413,830,032 weighted visits for children, 16.9% resulted in an antibiotic prescription. Among these visits, the unadjusted prevalence of inappropriate antibiotic prescriptions was 29.1% overall, 23.4% for safety-net patients, and 32.4% for privately insured patients (adjusted difference, safety-net minus private: -11.9%, 95% CI: -20.6%, -3.2%). Of 1,240,610,825 weighted visits for non-elderly adults, 11.0% resulted in an antibiotic prescription. Among these visits, the unadjusted prevalence of inappropriate antibiotic prescriptions was 50.1% overall, 60.7% for safety-net patients, and 47.9% for privately insured patients (adjusted difference, safety-net minus private: 11.3%, 95% CI: 2.4%, 20.3%).

**Conclusion:**

Safety-net status was associated with a lower rate of inappropriate antibiotic prescribing among children but a higher rate among non-elderly adults. Antibiotic stewardship initiatives targeting clinicians who care for privately insured children and adults with safety-net insurance may be warranted.

**Disclosures:**

**Jeffrey A. Linder, MD, MPH, FACP**, Amgen, Biogen, Eli Lily: Stocks/Bonds

